# The Effect of Plasma Rich in Growth Factors on Microglial Migration, Macroglial Gliosis and Proliferation, and Neuronal Survival

**DOI:** 10.3389/fphar.2021.606232

**Published:** 2021-02-26

**Authors:** Noelia Ruzafa, Xandra Pereiro, Alex Fonollosa, Javier Araiz, Arantxa Acera, Elena Vecino

**Affiliations:** ^1^ Experimental Ophthalmo-Biology Group (GOBE, www-ehu.eus/GOBE), Department of Cell Biology and Histology, University of Basque Country UPV/EHU, Leioa, Spain; ^2^ Begiker-Ophthalmology Research Group, BioCruces Health Research Institute, Cruces Hospital, Bilbao, Spain; ^3^ Department of Ophthalmology, University of Basque Country UPV/EHU, Leioa, Spain; ^4^ Biodonostia Health Research Institute, Donostia Hospital, San Sebastian, Spain

**Keywords:** retina, ophthalmology, plasma rich in growth factors, microglia, inflammation, cytokines, neuron, glia

## Abstract

Plasma rich in growth factors (PRGF) is a subtype of platelet-rich plasma that has being employed in the clinic due to its capacity to accelerate tissue regeneration. Autologous PRGF has been used in ophthalmology to repair a range of retinal pathologies with some efficiency. In the present study, we have explored the role of PRGF and its effect on microglial motility, as well as its possible pro-inflammatory effects. Organotypic cultures from adult pig retinas were used to test the effect of the PRGF obtained from human as well as pig blood. Microglial migration, as well as gliosis, proliferation and the survival of retinal ganglion cells (RGCs) were analyzed by immunohistochemistry. The cytokines present in these PRGFs were analyzed by multiplex ELISA. In addition, we set out to determine if blocking some of the inflammatory components of PRGF alter its effect on microglial migration. In organotypic cultures, PRGF induces microglial migration to the outer nuclear layers as a sign of inflammation. This phenomenon could be due to the presence of several cytokines in PRGF that were quantified here, such as the major pro-inflammatory cytokines IL-1β, IL-6 and TNFα. Heterologous PRGF (human) and longer periods of cultured (3 days) induced more microglia migration than autologous porcine PRGF. Moreover, the migratory effect of microglia was partially mitigated by: 1) heat inactivation of the PRGF; 2) the presence of dexamethasone; or 3) anti-cytokine factors. Furthermore, PRGF seems not to affect gliosis, proliferation or RGC survival in organotypic cultures of adult porcine retinas. PRGF can trigger an inflammatory response as witnessed by the activation of microglial migration in the retina. This can be prevented by using autologous PRGF or if this is not possible due to autoimmune diseases, by mitigating its inflammatory effect. In addition, PRGF does not increase either the proliferation rate of microglial cells or the survival of neurons. We cannot discard the possible positive effect of microglial cells on retinal function. Further studies should be performed to warrant the use of PRGF on the nervous system.

## Introduction

Platelet preparations, including platelet-rich plasma (PRP), contain high concentrations of growth factors that promote regeneration, and they are generally considered to be safe to use and typically inexpensive to obtain (Marx et al., 1998; Wang and Avila, 2007; Cole et al., 2010; Dhurat and Sukesh, 2014). Plasma rich in growth factors (PRGF) is a subtype of P-PRP (pure platelet-rich plasma), which are preparations without leukocytes and with a low-density fibrin network that may stimulate tissue regeneration (Dohan Ehrenfest et al., 2014). Autologous PRGF has been approved for clinical use in the European Community and by the U.S. Food and Drug Administration (FDA: Hayashi and Takagi, 2015; Cui et al., 2017; Osborne et al., 2018), and it has commonly been employed in ophthalmology in the form of eye drops (Lopez-Plandolit et al., 2010; Lopez-Plandolit et al., 2011; Merayo-Lloves et al., 2015; Sanchez-Avila et al., 2018). In addition, pilot studies are currently being carried out regarding the use of PRGF in retinal surgery to treat persistent macular holes (Arias et al., 2019) and autologous platelet injections have been used to treat recurrent retinal detachment (Nadal et al., 2012). However, the full range of responses that could be triggered by PRGF are not fully understood.

The PRGF used in the clinic is often autologous, obtained from the patient’s own blood, although human PRGF has also been used in studies with mice (Anitua et al., 2013; Anitua et al., 2014b; Anitua et al., 2015a). This fact could be important to establish therapies for patients suffering from autoimmune diseases whose autologous PRGF may contain auto-antibodies (Anitua et al., 2014a), or for patients in whom their own PRGF is not as effective as expected due to variations in specific factors (Vahabi et al., 2015). Thus, we set out here to assess the effect of PRGF of two different origins. The model animal selected for this study was the pig, as the porcine retina is the most similar to the human retina among large mammals (Prince and Ruskell, 1960; Vecino and Sharma, 2011).

In the central nervous system (CNS), PRGF can interact with glial cells, promoting their survival, proliferation and differentiation (O’kusky et al., 2000; Jin et al., 2002; Anitua et al., 2009; Anitua et al., 2014b), as well as influencing inflammatory processes (Anitua et al., 2015a). Microglia are one of the three main types of glial cells in the mammalian retina and they form a population of resident macrophages within the CNS that are not only involved in immune responses but also, that participate in the development and maintenance of neural networks. Thus, microglia represent an important nexus between the neurological and immunological activity in the CNS (Fernandes et al., 2014; Gertig and Hanisch, 2014; Vecino et al., 2016; Davis et al., 2017).

Microglia are quiescent in the normal adult retina, exhibiting a characteristic ramified morphology that allows them to scan their environment and phagocytose cell debris. In these conditions, resident microglia are strictly limited to the inner retinal layers, mainly populating the plexiform layers (Lee et al., 2008; Buschini et al., 2011). However, microglia become activated in response to neuronal injury or immunological stimuli (Streit et al., 1988; Kreutzberg, 1996; Block and Hong, 2005) and in the retina, the activation of resident microglia drives their migration to the outer retinal layers, where they transform into activated, ameboid, full-blown phagocytes that interact with infiltrating blood cells (Karlstetter et al., 2015). All types of neuroinflammation produce chronic microglial activation, including ocular inflammation, which is coupled to the release of inflammatory mediators and phagocytosis (Block and Hong, 2005). Thus, activated microglia fulfill an important role in the initiation and propagation of neurodegenerative processes (Madeira et al., 2015a). Given the location and size of microglia, and the fact that they may migrate to the outer layers of the retina in an inflammatory explant model, these cells resemble the hyperreflective dots that can be seen in the ONL and external limiting membrane in uveitis and ocular inflammation when studied by optical coherence tomography (OCT) *in vivo* (Coscas et al., 2013; Vujosevic et al., 2013; Pakzad-Vaezi et al., 2014). These dots are a sign of disease progression and prognosis, such that the *in vivo* imaging of microglia by OCT may increase its diagnostic value in clinical ophthalmology, and this can be studied in the model of organotypic retinal culture used here. Therefore, we analyzed the inflammatory response to PRGF by assessing microglial cell migration in retinal explants exposed to PRGF and by characterizing the inflammatory cytokines it contains. Moreover, the inflammatory response to PRGF was compromised by heat inactivation, by adding dexamethasone (Dex) as an anti-inflammatory drug, or by adding antibodies against some pro-inflammatory cytokines to the PRGF in order to study whether these manipulations inhibit the inflammatory response of microglia to PRGF.

In relation to the inflammatory processes, macroglia also respond to and undergo reactive gliosis. Retinal macroglia include Müller cells and astrocytes, the former extending across the thickness of the retina to provide structural stability and maintaining close contact with the majority of retinal neurons. Astrocytes are mostly located in the nerve fiber layer and they accompany the blood vessels. Both these cell types provide trophic factors to neurons, which promote cell survival and repair, as well as maintaining retinal homeostasis and that of the blood–retina barrier (Bringmann and Reichenbach, 2001; Bringmann et al., 2006; Reichenbach and Bringmann, 2013; Vecino et al., 2016). Reactive gliosis is a response to a multitude of insults and disorders, altering the thickening and enlargement of Müller cells, and of astrocyte processes. Typical features of macroglial gliosis involve cellular hypertrophy, such as the up-regulation of intermediate filament (IF) expression (e.g., glial fibrillary acidic protein -GFAP) or increased rates of proliferation (Barres, 2008; Vecino et al., 2016). Thus, the rate of glial cell proliferation may be altered in the presence of PRGF as it is known to contain growth factors that accelerate glial cell proliferation (O’kusky et al., 2000; Jin et al., 2002; Anitua et al., 2009; Anitua et al., 2014b), such as platelet-derived growth factor (PDGF), fibroblast growth factor (FGF), epidermal growth factor (EGF) and nerve growth factor (NGF: Orive et al., 2009; Anitua et al., 2010). Therefore, as PRGF may induce an inflammatory response in the retina, we set out to determine whether PRGF stimulates gliosis and the proliferation of glial cells in retinal explants. In cultured Müller cells, we have demonstrated the capacity of PRGF to increase the number of the Müller glia (Ruzafa et al., 2021).

Finally, an intranasal delivery system of PRGF has been designed in order to reverse the neurodegeneration in a transgenic mouse model of Alzheimer’s disease (AD: Suga et al., 2014; Osborne et al., 2018), based on PRGF acting as a neurotrophic factor promoting neuronal survival in the presence of amyloid-β (Suga et al., 2014; Pan et al., 2019). The retina, as part of the CNS, has a limited capacity for repair after disease or lesion, and retinal lesions cause the death of retinal ganglion cells (RGCs), the neurons responsible for the communication between the eye and brain. These lesions might result in irreversible blindness (Newman and Reichenbach, 1996; Garcia et al., 2002), as occurs in the case of glaucoma (Glovinsky et al., 1991; Wygnanski et al., 1995), axonal degeneration (Newman and Reichenbach, 1996) or ischemia (Selles-Navarro et al., 1996; Nadal-Nicolas et al., 2012). However, RGCs can recover their regenerative capacities in appropriate environments (Fischer and Reh, 2003; Berry et al., 2008). Thus, and based on the potential neuroprotective properties of PRGF, we finally set out to determine whether PRGF affects the survival of RGCs.

In summary, we examined here the effect of PRGF of two different origins in organotypic retinal cultures, analyzing microglia migration, gliosis, proliferation and RGC survival. The inflammatory cytokines in PRGF were also quantified and the suppression of its inflammatory response was also studied.

## Materials and Methods

### Study Design

**FIGURE 1:**
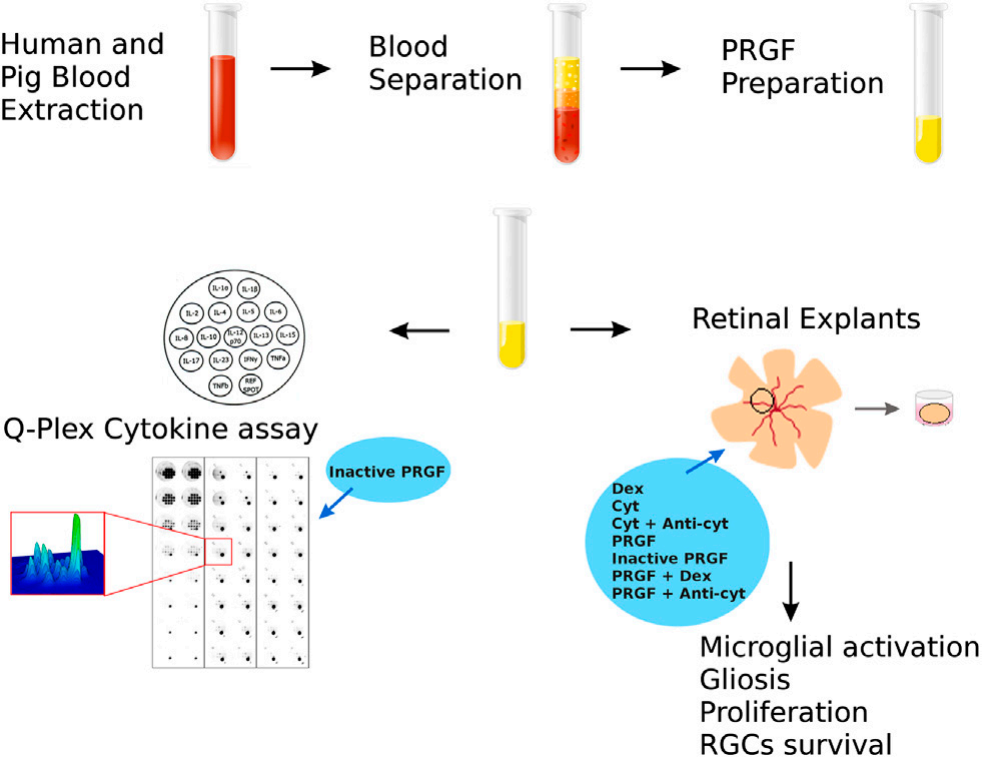
Scheme to analyze the properties of the PRGF in organotypic cultures of adult porcine retinas. First, PRGF was obtained from human and porcine blood by separation and plasma extraction. Subsequently, the effect of the PRGF was evaluated on retinal explants by assessing microglial activation, gliosis and proliferation, and RGC survival. In addition, the cytokines in the human and porcine PRGF was quantified using a Q-plex assay. Finally, given the implication of PRGF in promoting inflammation, the effects of exposing the explants exposed to dexamethasone (Dex), cytokines (Cyt: IL-1β, IL-6 and TNFα) or cytokines plus antibodies raised against these cytokines (anti-cyt: anti-IL-1β, IL-6 and TNFα) were also evaluated. Finally, we examined the effect of heat-inactivated PRGF, as well as PRGF combined with Dex or anti-cyt, analyze their effects on microglial migration.

To analyze the effect of PRGF in the retina, we used retinal explants to assess the inflammatory effect of PRGF and the migration of microglial cells in these explants (see Figure 1). In addition, the effect of PRGF on the proliferation, gliosis and survival of RGCs was also assessed in these organotypic retinal cultures, as were the cytokines present in the PRGF.

### Porcine Samples

Adult porcine eyes (n = 20) and blood (n = 5) were obtained from a local slaughterhouse and the eyes were transported to the laboratory on ice in CO_2_-independent medium (Life Technologies, Carlsbad, CA, USA) plus 0.1% gentamicin. The retinas were dissected out of the eyes 1–2 h after enucleation. All animal experimentation adhered to the ARVO Statement for the Use of Animals in Ophthalmic and Vision Research.

### Human and Pig Plasma Rich in Growth Factors

Qualified personnel carried out this research and approval for the study was obtained in strict accordance with the tenets of the Helsinki Declaration on Biomedical Research Involving Human Subjects. Before blood collection, signed informed consent was obtained from all the subjects once the nature of the study and the possible consequences of the study had been explained. Human blood samples were obtained through antecubital vein puncture and PRGF was obtained as described previously (Suga et al., 2014), with some minor modifications. Briefly, human (n = 3) and porcine (n = 5) blood was collected into 5 ml tubes containing 3.8% (wt/vol) sodium citrate. Samples were centrifuged at 460 g for 8 min at room temperature and the plasma fraction containing platelets but not the buffy coat and erythrocytes were separated. The plasma fraction (1 ml) was reconstituted for 1 h at 34°C with 50 µL calcium chloride (Braun Medical, Melsungen, Germany) in glass tubes, and the supernatant released was collected after centrifugation at 460 g for 15 min. Finally, part of the total volume of the PRGF obtained was heat-inactivated at 56°C for 60 min following a protocol published previously (Anitua et al., 2014a), and both the samples (PRGF and inactive PRGF) were filtered through a filter pore size of 0.2 mm (Fisher Scientific, Madrid, Spain), aliquoted and stored at −80°C.

### Organotypic Retinal Cultures

Under aseptic conditions, each pig eyeball was immersed in 70% ethanol, washed in clean CO_2−_independent medium and neuroretinal explants were obtained as described previously (Del Rio et al., 2011). After removing the lens and the vitreous humor, the entire retina was detached by paint brushing and cutting the optic nerve. Using an 8 mm diameter dissecting trephine, five retinal explants were obtained from the middle part of the retina of each eye, at the same distance from the optic nerve and excluding the larger vessels. The explants were transferred to cell culture inserts (0.45 μm pore, 12 mm diameter: Merck Millipore, Darmstadt, Germany) with the photoreceptor layer facing the membrane, and they were cultured in Neurobasal A medium (Life Technologies, Carlsbad, CA, USA) supplemented with 1% L-glutamine (2 mM: Life Technologies, Carlsbad, CA, USA) and 0.1% gentamicin (50 mg/ml: Life Technologies, Carlsbad, CA, USA), and they were maintained at 37°C in a humid atmosphere of 5% CO_2_ for 1 and 3 days. The culture medium was maintained in contact with the support membrane beneath the explant and changed with freshly prepared warmed medium every other day. To study the inflammatory role of the PRGF, explants were cultured in the different experimental conditions (Table 1) and at least three replicates from different animals were used for each condition. No more than one explant from the same eye was used for each experimental condition.

**TABLE 1:** Different experimental conditions in which the explants were exposed to PRGF.

Control (0% PRGF)
Dex
Cytokines
Cytokines + anti-cytokines
10% autologous pig PRGF
10% heterologous pig PRGF
10% human PRGF
10% inactive human PRGF
10% human PRGF + dex
10% human PRGF + anti-cytokines

The explants were cultured in the presence or absence of 10% PRGF from the beginning of the organotypic cultures, using PRGF obtained from the same animal (autologous), from another pig (heterologous) or from a human donor. The human PRGF was added in either an active or inactive form. Dexamethasone (Dex, 1 μM: Sigma Aldrich, St. Louis, MO, USA) was also added to the explants or a mixture of three pro-inflammatory cytokines: IL-1β, IL-6 (10 ng/ml) and TNFα (20 ng/ml, as recommended in Madeira et al., 2015b: Sigma-Aldrich, St. Louis, MO, USA). In another experimental condition these cytokines were inactivated by adding antibodies against the three cytokines (1 μg/ml, as recommended in Madeira et al., 2015b): goat anti-IL-1β and mouse anti-IL-6 (R&D System, Minneapolis, MN, USA); and rabbit anti-TNFα (Peprotech, London, United Kingdom) antibodies). In both cases, the cytokines were also added from the beginning of the culture of the explants with or without their inhibitors. The explants were cultures for 1 day and for 3 days. On the other hand, to study the effect of PRGF on gliosis, proliferation and RGC survival, selected explants were cultured for 3 days in the presence of 10% human PRGF, which induced a stronger effect on microglia.

At the end of each experiment, the retinal explants were fixed overnight in 4% paraformaldehyde (PFA), cryoprotected overnight at 4°C in 30% sucrose in 0.1 M PB (phosphate buffer, pH 7.4), and they were then embedded in Tissue-Tek O.C.T. Compound to obtain cryosections (14 μm) that were stored at −20°C.

### Immunochemistry

The cryostat sections of the explants were immunostained to detect microglia, gliosis, proliferative cells and RGCs, as described previously (Vecino et al., 2002). The sections were washed twice for 10 min with PBS (phosphate buffered saline, pH 7.4) containing Triton X-100 (0.25%, TX) and they were incubated overnight with primary antibodies in PBS-TX containing BSA. The primary antibodies used were an: anti-Iba1 rabbit antibody (1:2,000, WAKO, Osaka, Japan), anti-GFAP mouse antibody (1:1,000, Sigma, Steinheim, Germany), anti-Ki67 rabbit antibody (1:100, Abcam, Cambridge, England), and anti-Brn3a goat antibody (1:1,000, Santa Cruz Biotechnology, Santa Cruz, USA). After two washes with PBS, the sections were incubated for 1 h with a secondary antibodies at a dilution of 1:1,000 in PBS-BSA (1%), and with a 1:10,000 dilution of the DAPI nuclear marker (Life Technologies, Carlsbad, AC, USA). The secondary antibodies used were anti-rabbit Alexa Fluor 555 or 488, anti-mouse Alexa Fluor 488 and anti-goat Alexa Fluor 568 (Life Technologies, Carlsbad, AC, USA). The sections were washed twice with PBS for 10 min and mounted with PBS-Glycerol (1:1) before observation under an epifluorescence microscope.

### Cells Quantification

Microglia were analyzed in at least three slides containing a minimum of six cryostat sections from each explant. The total extension of the retina analyzed was approximately 10 linear centimetres and this procedure was carried out on at least three replicates for each condition. The total number of microglia cells (Iba1) was manually counted and the number of microglia per linear mm of retina was calculated. In addition, the number of microglia in the outer nuclear layer (ONL) was also counted and the proportion of microglia in this layer of the retina was calculated for each condition. Similarly, RGC density was also analyzed, calculated as the mean number of RGCs (Brn3a^+^) per linear mm of retina. Cell quantification and images of Müller and astrocyte gliosis (GFAP), or images of proliferative cells (Ki67), were obtained on an epifluorescence microscope (Zeiss, Jena, Germany) coupled to a digital camera (Zeiss Axiocam MRM, Zeiss, Jena, Germany), and using the Zeiss Zen software (Zeiss, Jena, Germany).

### Multiplex Cytokine Assays

To detect and quantify the cytokines present in the different PRGF samples, a multiplex enzyme-linked immunosorbent assay (ELISA) was used to measure: IL-1α, IL-1β, IL-2, IL-4, IL-5, IL-6, IL-8, IL-10, IL-12p70, IL-13, IL-15, IL-17, IL-23, IFNγ, TNFα, and TNFβ (Q-Plex™, Human Cytokine Screen, 110996HU, Quansys Bioscience, Logan, UT, USA). The assay was performed according to the manufacturer´s instructions, analyzing 100 μL of the different human and porcine PRGF samples (pig, inactive pig, human, inactive human and human plus Dex) in each well of a 98-well plate Q-Plex™. The standards were measured in duplicate and cytokine concentrations were calculated using a standard curve. Each individual sample was assessed in four experimental replicates and the arithmetic averages were calculated.

### Statistical Analyses

Statistical analyses were carried out using the SPSS software v. 21.0 (IBM), and the mean and standard error was calculated for each condition. The data from the different experimental conditions were compared using an analysis of variance (ANOVA), followed by the Games-Howell test as there was no homogeneity of the variances according to a Levene test. For the multiplex cytokine assays, a non-parametric test was used, the Kruskal-Wallis H test. The minimum value for significant differences in all the tests was defined as *p* < 0.05.

## Results

### Microglial Migration in Retinal Organotypic Cultures in the Presence of Plasma Rich in Growth Factors

**FIGURE 2:**
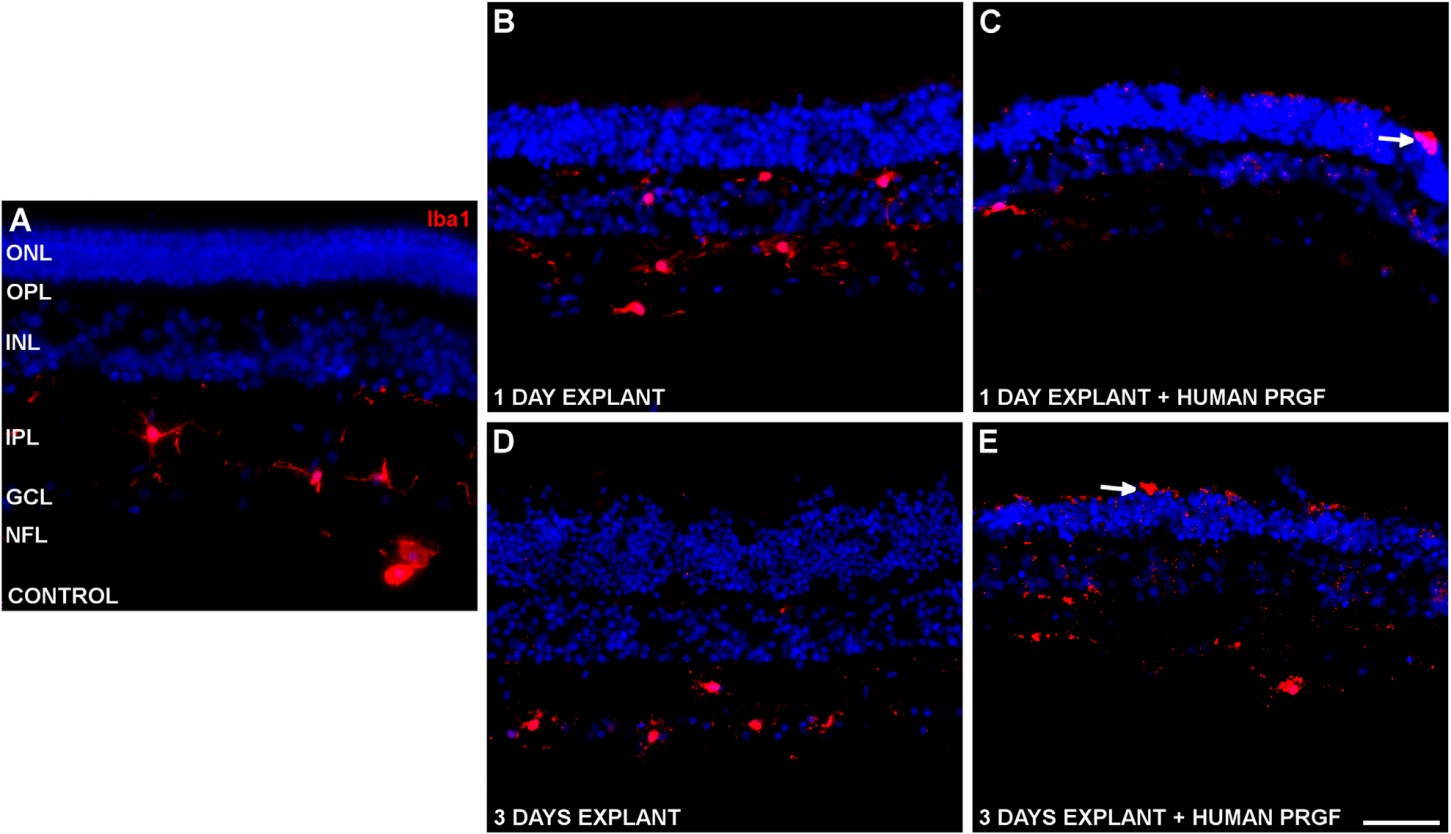
Microglia in a control retina and in organotypic retinal cultures maintained in the presence or absence of human PRGF. Representative images of an adult pig retina from a control eye **(A)**, or of explants of organotypic cultures maintained in the absence **(B,D)** or in the presence **(C,E)** of 10% human PRGF. The explants were cultured for 1 **(B,C)** or 3 **(D,E)** days, labeling the microglial cells with antibodies against Iba1 (red, anti-Iba1 rabbit antibody, 1:2,000, WAKO, Osaka, Japan) and the nuclei with DAPI (blue). The white arrows point to microglial cells in the ONL: ONL, outer nuclear layer; OPL, outer plexiform layer; INL, inner nuclear layer; IPL, inner nuclear layer; GCL, ganglion cell layer; NFL, nerve fiber layer. Scale bar = 100 μm.

Changes in microglial morphology were evident in the retinal explants (Figure 2), these cells adopting an ameboid shape distinct to the ramified morphology observed in the control retina. However, there were no changes in the total number of microglial cells, with an average of 12.24 ± 0.25 microglial cells/mm. These microglia were generally located in the inner part of the retina (Figure 2A), yet the microglia were able to migrate to the outer plexiform layer (OPL) when maintained in the absence of PRGF during the culture (Figures 2B,D). However, these cells migrated to the ONL only when the retinal explants were cultured in the presence of PRGF (Figures 2C,E).

**FIGURE 3:**
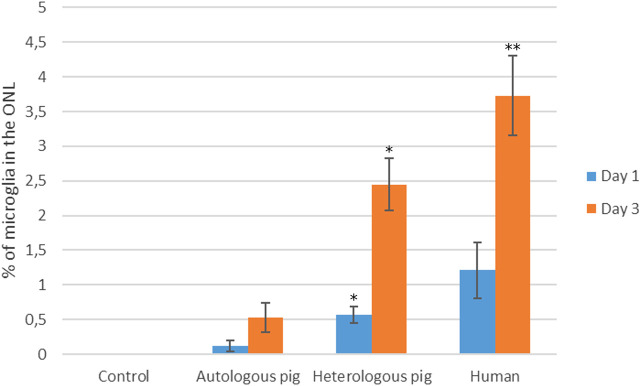
Quantification of microglia migration in the presence of autologous and heterologous porcine PRGF or human PRGF. Microglial migration on day 1 and 3 in retinal explants treated with 10% PRGF from the same pig as the retinal explant (autologous) (n = 5), from another pig (heterologous) (n = 5) or from human blood (n = 5). Microglial migration is represented as the percentage of microglial cells located in the outer nuclear layer (ONL) relative to the total number of microglia: *p < 0.05, **p < 0.01 relative to the control.

Since microglial migration is a sign of activation, the proportion of microglia that migrated to the ONL was quantified. Initially, the migration of microglia was analyzed in distinct retinal explants treated with 10% PRGF (Figure 3).

On day 1, the proportion of microglia in the ONL of the retinal explants maintained in the presence of 10% PRGF was 0.12 ± 0.07% when autologous PRGF was used, 0.56 ± 0.11% with heterologous pig PRGF and 1.21 ± 0.4% for human PRGF, and on day 3 these values increased to 0.53 ± 0.22%, 2.45 ± 0.38% and 3.73 ± 0.57%, respectively. There were no significant differences between the control explants, in which there was no microglial migration to the ONL, and those maintained in the presence of autologous pig or human PRGF at day 1. However, by day 3 this parameter was significantly different in explants maintained in the presence of heterologous pig PRGF (*p* < 0.05) and human PRGF (*p* < 0.01) relative to the controls (the specific *p*-values are given in the Supplementary Material).

### Quantification of the Cytokines in Plasma Rich in Growth Factors

Having shown that PRGF can activate microglial cells and promote their migration, we analyzed the inflammatory cytokines present in the pig and human PRGF, and in the heat-inactivated pig and human PRGF. In the different PRGF samples analyzed, we confirmed the presence of and quantified the following cytokines: IL-1α, IL-1ß, IL-2, IL-4, IL-6, IL-8, IL-10, IL-12, IL13, IL-15, IL-17, IL-23, TNFα, and TNFβ (Table 2).

**TABLE 2:** Concentration (pg/ml) of the cytokines in porcine and human PRGF

Cytokine	Pig PRGF	Inactive pig PRGF	Human PRGF	Inactive human PRGF
IL-1α	8.15 ± 1.67	10.31 ± 0.87	6.34 ± 2.18	6.55 ± 2.39
IL-1ß	14.65 ± 4.93	20.91 ± 7.06	17.03 ± 4.35	17.01 ± 2.54
IL-2	4.58 ± 1.15	6.93 ± 0.13	4.54 ± 0.54	6.17 ± 1.66
IL-4	0.29 ± 0.28	0.87 ± 0.70	0.39 ± 0.32	0.17 ± 0.17
IL-6	3.61 ± 0.49	5.21 ± 0.18	4.24 ± 1.08	3.99 ± 1.14
IL-8^a^	1.32 ± 0.42	1.24 ± 0.05	29.59 ± 5.73	29.21 ± 3.38
IL-10	4.65 ± 1.47	6.85 ± 2.65	7.21 ± 1.52	4.73 ± 1.61
IL-12	4.96 ± 1.11	5.86 ± 2.11	5.00 ± 3.87	6.71 ± 3.90
IL-13	0.37 ± 0.21	0.29 ± 0.22	0.75 ± 0.75	0.35 ± 0.20
IL-15	2.29 ± 0.49	4.54 ± 1.23	7.09 ± 2.79	6.77 ± 1.92
IL-17	4.59 ± 0.21	2.93 ± 1.02	1.68 ± 0.99	2.88 ± 0.97
IL-23	185.47 ± 43.63	242.71 ± 86.04	283.14 ± 214.27	222.54 ± 158.70
TNFα	4.65 ± 4.25	3.76 ± 3.76	6.61 ± 2.99	1.84 ± 1.84
TNFß	18.40 ± 4.64	14.07 ± 9.76	18.15 ± 14.21	14.88 ± 13.96

^a^ significant differences between pig (n = 5) PRGF and human PRGF (n = 3).

When comparing the cytokine concentrations in pig and human PRGF, IL-8 was the only cytokine detected at a significantly higher concentration in the human than in the pig PRGF (*p* < 0.001). In terms of the heat-inactivated pig and human PRGFs, no differences were found in the cytokine concentrations relative to the PRGF in either species, indicating that this heat-inactivation does not affect the cytokine concentrations (the cytokines activity was not assessed). However, we did appreciate some variability in the quantification of cytokines and thus, we analyzed the different cytokine concentrations between individuals. Significant differences in the cytokine concentrations of human PRGF were found between individuals: IL-8, IL-12, IL-13, IL-15, IL-17, IL-23, and TNFα (*p* < 0.05). Significant differences among the cytokines in pig PRGF were also found between different individuals: IL-1ß, IL-10, IL-15, IL-17, and IL-23 (*p* < 0.05). The remaining cytokines were homogeneously distributed between individuals and no significant differences were found between them (the specific *p*-values are given in the Supplementary Material).

### Suppression of Microglial Migration in the Presence of Plasma Rich in Growth Factors

**FIGURE 4:**
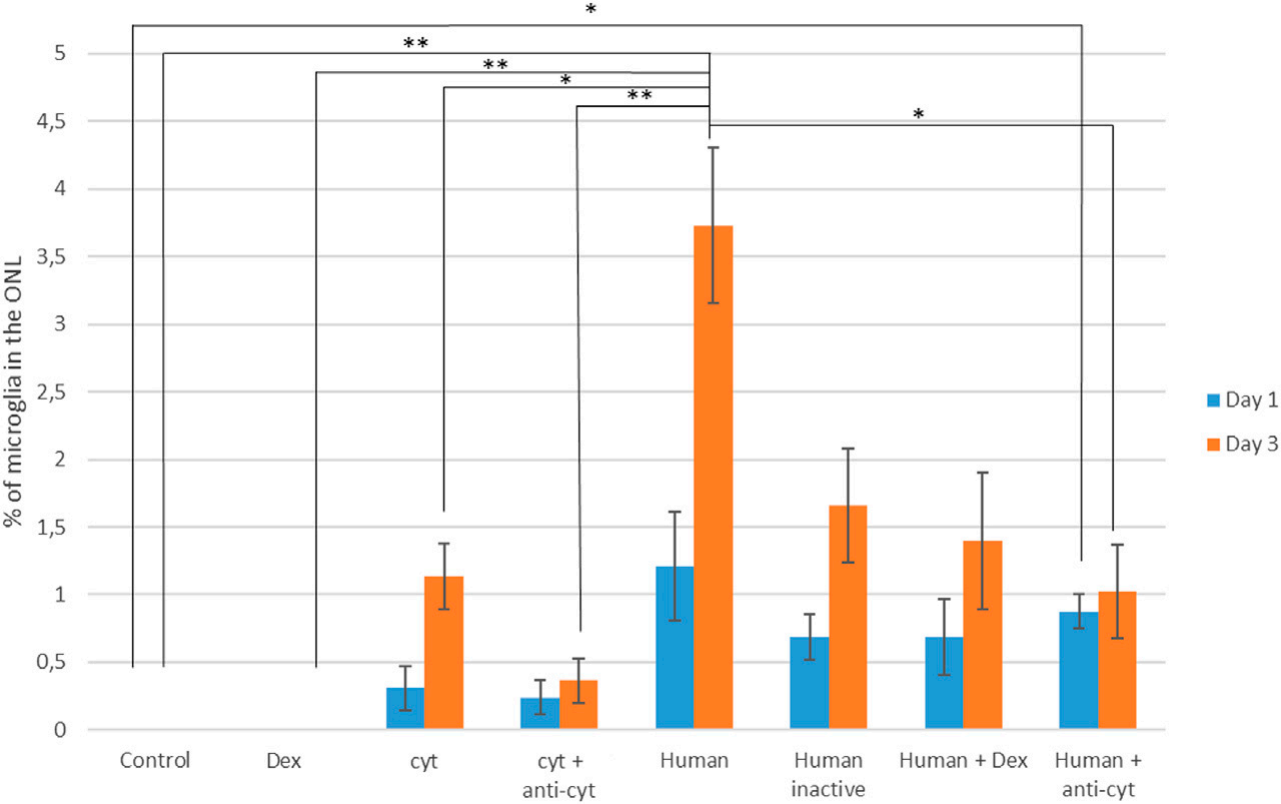
Quantification of microglial migration in the presence of human PRGF, inhibiting its inflammatory effect. Microglial migration is represented as the proportion of microglial cells located in the outer nuclear layer (ONL) relative to the total number of microglia, reflecting the migration in control conditions (n = 5), and in the presence of dexamethasone (Dex: n =), cytokines (cyt, IL-1β, IL-6 and TNFα: n = 4), cytokines plus anti-cytokine antibodies (anti-cyt: n = 3), with 10% human PRGF (Human: n = 5). The inflammatory effect of PRGF (Human) on microglial migration was inhibited by PRGF heat-inactivation (Human inactive: n = 5), or by adding Dex (n = 5) or anti-cytokine antibodies (n = 5). Significant differences relative to the control conditions and the presence of human PRGF are shown: *p < 0.05, **p < 0.01.

The suppression of microglia migration was assessed in retinal explants maintained in the presence of 10% human PRGF, conditions that induced a higher proportion of microglial migration. As such, we analyzed the microglial migration in explants maintained under different culture conditions: adding cytokines or blocking them with antibodies; adding human PRGF, heat-inactivating it or adding antibodies against cytokines; or adding Dex alone or in combination with PRGF (Figure 4). To test the role of cytokines, we added a mixture of three major pro-inflammatory cytokines (IL-1β, IL-6 and TNFα) alone or in combination with antibodies raised against these cytokines. The microglia migration in the explants in the presence of cytokines was 0.31 ± 0.16% on day 1 and 1.14 ± 0.25% on day 3, which fell to 0.24 ± 0.12% on day 1 and 0.37 ± 0.16% on day 3 in the presence of antibodies against these cytokines. However, in none of these conditions were significant differences produced relative to the control conditions. Fewer microglia migrated in the presence of PRGF and the anti-cyt mixture, 0.88 ± 0.12% on day 1 and 1.02 ± 0.34% on day 3, and the latter figure in presence of the cytokine antibodies was significantly lower (*p* < 0.05) than in the explants treated with 10% of human PRGF alone on day 3.

The percentage of microglia in the ONL on day 1 was 1.21 ± 0.4% when the explants were exposed to 10% human PRGF and 3.73 ± 0.57% on day 3. When this PRGF was heat-inactivated, microglial migration decreased to 0.68 ± 0.16% on day 1 and 1.65 ± 0.42% on day 3. In the presence of Dex and PRGF, microglial migration also decreased to 0.69 ± 0.28% on day 1 and 1.29 ± 0.5% on day 3. However, this apparent decrease in microglial migration was not significantly different from the migration in the presence of 10% PRGF alone and nor was it significantly different from the values in the control explants. There was a reduction in microglial migration when the PRGF was heat-inactivated, or when it was used in combination with Dex or anti-cyt antibodies, although these conditions never completely suppressed migration. Thus, the pro-migratory effect of PRGF on microglia appears to be multifactorial and not only due to the influence of pro-inflammatory cytokines.

### Effect of Plasma Rich in Growth Factors on Gliosis and Proliferation in Organotypic Cultures

**FIGURE 5:**
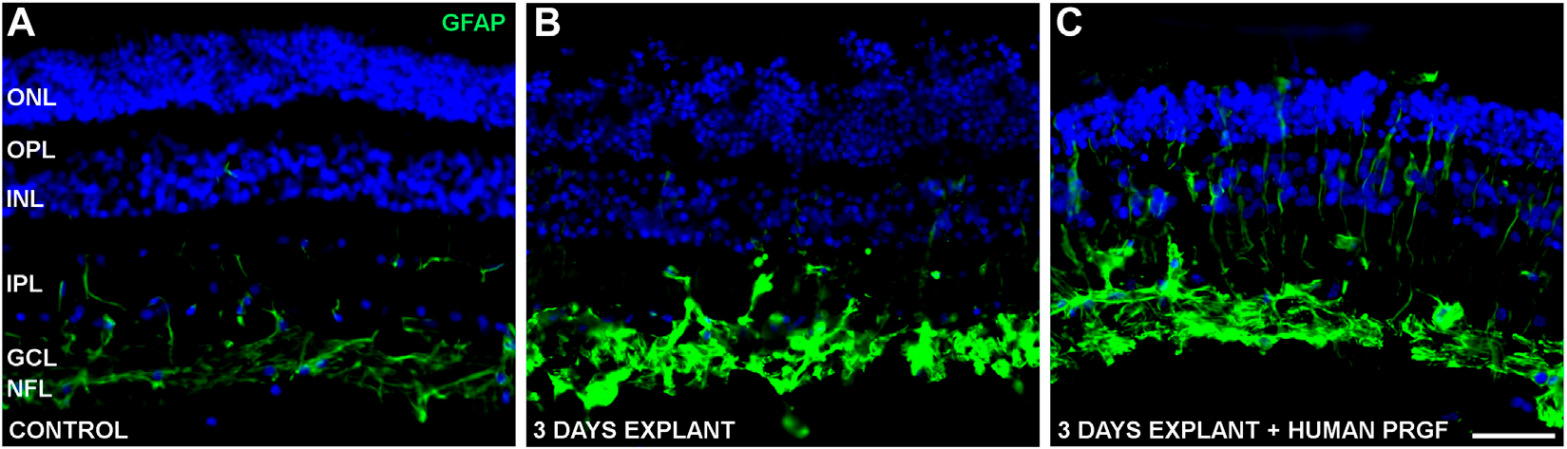
Gliosis in control retinas and in organotypic retinal cultures maintained in the presence or absence of human PRGF. Representative images of adult pig retinas from a control eye (n = 5: **A)**, or from explants of organotypic cultures maintained in the absence (n = 4: **B)** or presence (n = 4: **C)** of 10% human PRGF. The GFAP in astrocytes and Müller cells was immunolabeled (green, anti-GFAP mouse antibody, 1:1,000, Sigma, Steinheim, Germany), and the nuclei were labeled with DAPI (blue): ONL, outer nuclear layer; OPL, outer plexiform layer; INL, inner nuclear layer; IPL, inner nuclear layer; GCL, ganglion cell layer; NFL, nerve fiber layer. Scale bar = 100 μm.

To determine if PRGF may modify macroglial (astrocytes and Müller glia) and microglial cells, the distribution of the cytoskeletal protein GFAP was studied in the retinal explants to establish the extent of astrocyte and Müller cell activation. In the control cultures, GFAP labeled astrocytes were detected in the GCL and NFL, and some Müller cells were labeled for GFAP (vertical lines in IPL in Figure 5A). The presence of PRGF seemed to induce the gliosis of Müller cells in the retinal explants (Figure 5C), although the astrocytes were already activated in explants in the absence of PRGF (Figure 5B). Thus, the induction of astrocyte gliosis by PRGF cannot be confirmed.

**FIGURE 6:**
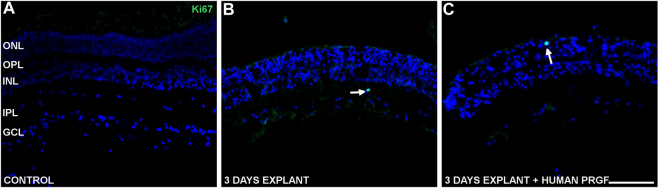
Proliferative cells in control retinas and in organotypic retinal cultures maintained in the presence or absence of human PRGF. Representative images of adult pig retinas from a control eye (n = 5: **A)**, and organotypic cultures maintained in the absence (n = 4: **B)** or presence (n = 4: **C)** of 10% human PRGF. The edge of the explant is also shown **(C)**. The proliferative cells were labeled with antibodies against Ki67 (green, anti-Ki67 rabbit antibody, 1:100, Abcam, Cambridge, England) and the nuclei with DAPI (blue). The white arrows indicate cells in an active phase of the cell cycle. The edge of the explant is shown to demonstrate the absence of proliferation (left in picture C): ONL, outer nuclear layer; OPL, outer plexiform layer; INL, inner nuclear layer; IPL, inner nuclear layer; GCL, ganglion cell layer. Scale bar = 100 μm.

To study the possible proliferation induced by PRGF we studied Ki67 as a marker of actively dividing cells. In control retinas, a few Ki67 labeled cells were detected in an active phase of the cell cycle (Figure 6), whereas in organotypic cultures proliferative cells were labeled with the anti-Ki67 antibody in the presence or absence of human PRGF (Figures 6B,C). These proliferative cells were rare, even at the edges of the explants (Figure 6C) and consequently, we cannot confirm that PRGF increases the rate of proliferation in organotypic cultures of adult retinas. Considering the position of the proliferative cells and given that only glial cells can divide in the retina, we conclude that these proliferative cells were microglia.

### Retinal Ganglion Cells Survival in Organotypic Cultures in the Presence of Plasma Rich in Growth Factors

**FIGURE 7:**
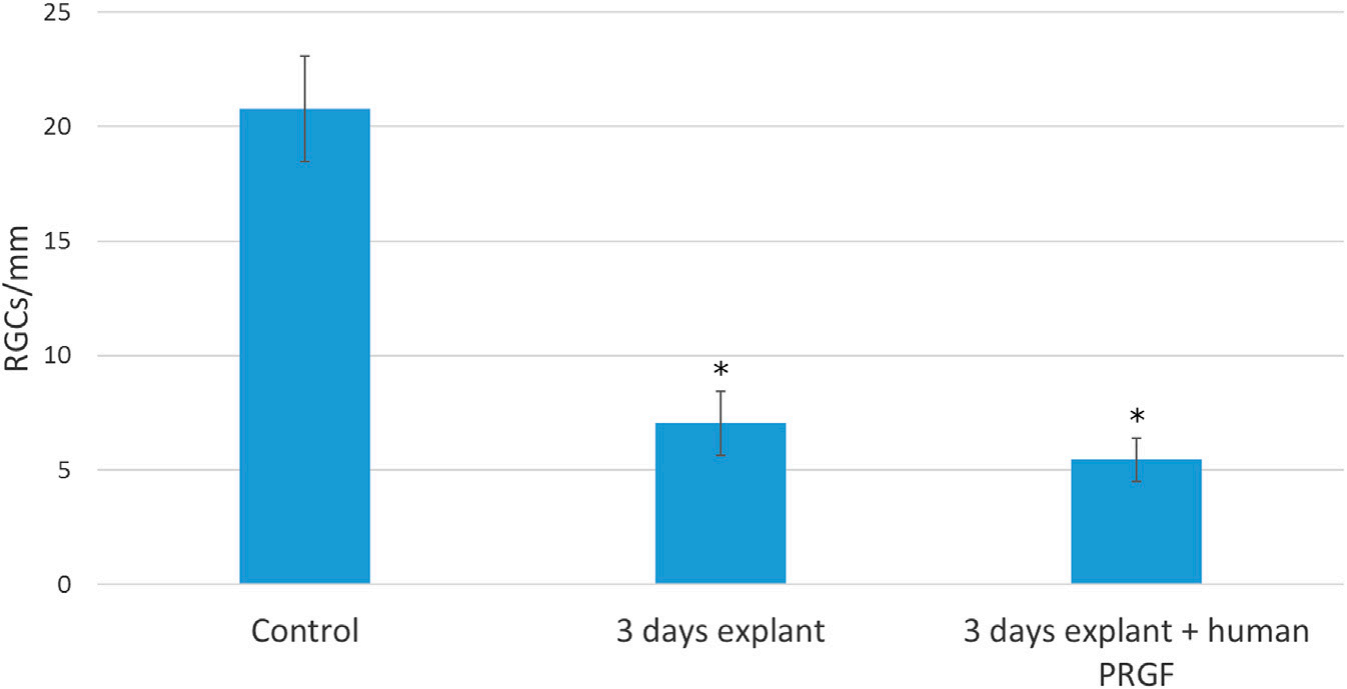
Quantification of RGCs per millimeter of retina in controls retinas and in explants maintained in the presence or absence of human PRGF. The number of RGCs per linear mm of retina are shown in control retinas (n = 5) and in organotypic cultures at day 3, maintained in the presence (n = 4) or absence of 10% human PRGF (n = 4). Significant differences between the controls and explants are indicated: *p < 0.05.

Finally, we analyzed the survival of retinal neurons, specifically RGCs (Figure 7), and relative to the control retinas (20.76 ± 2.3 RGCs/mm), there were significant fewer RGCs in explants maintained in either the presence (5.44 ± 0.95 RGCs/mm) or absence (7.05 ± 1.41 RGCs/mm) of human PRGF. However, we did not find a significant difference in the survival of RGCs in organotypic cultures maintained in the presence of PRGF and thus, PRGF does not appear to offer neuroprotection to RGCs in explants of adult porcine retinas (the specific *p*-values are given in the Supplementary Material).

## Discussion

PRGF is a biological supplement which provides a pool of growth factors that can stimulate and accelerate tissue regeneration, and hence, it may potentially represent a therapeutic agent to treat different diseases, including neurodegenerative diseases (Anitua et al., 2013; Anitua et al., 2014b; Anitua et al., 2015a). In this study, the influence of PRGF on glial cells and CNS neurons was assessed in the retina. In organotypic retinal cultures, PRGF promotes the migration of microglia, a sign of inflammation that could be due to the cytokines it contains. However, PRGF does not induce gliosis among astrocytes and partially in Müller glia, and it does not alter the proliferation of glial cells or modify RGC survival.

One of the critical steps in the inflammatory response is the recruitment and migration of immune and inflammatory cells to inflammatory sites, and in the CNS, microglia are the resident innate immune cells (Lull and Block, 2010; Omri et al., 2011). In a healthy retina, microglia are located in the inner part of retina (Streit et al., 1988; Ling and Wong, 1993; Omri et al., 2011) but when these cells are activated, they migrate into the sub-retinal space, as occurs in several retinopathies associated with ocular inflammation like diabetic retinopathy (Zeng et al., 2008) and age-related macular degeneration (Combadiere et al., 2007). Microglial cells were analyzed in organotypic cultures or explants, a commonly used model of inflammation (Carter and Dick, 2003; Bauer et al., 2016), assessing the effect of PRGF. In addition to the microglial migration observed, we detected a change in their morphology from a ramified to an ameboid shape in the cultured retinas.

It has been suggested that PRGF could fulfill an inherent anti-inflammatory role, mediated by NF-kB (Anitua et al., 2014b; Anitua et al., 2015a). In addition, PRGF could dampen the inflammatory status of a cell culture model of inflammation induced by IL-1β and TNFα, significantly decreasing ICAM-1 and COX-2 levels when compared to autologous serum (Anitua et al., 2016). Thus, to understand how PRGF activates retinal microglia, the presence of inflammatory cytokines in the PRGF was quantified. It has been suggested that pro-inflammatory cytokines are almost absent or limited in PRGF (Anitua et al., 2015b), although some cytokines have been detected in PRGF: IL-6 (Masuki et al., 2016), IL-8 (Anitua et al., 2010), IL-4, and TNFα (Anitua et al., 2012). The concentrations of the cytokines that were observed in PRGF may be lower than that of some interleukins in other PRPs (Pochini et al., 2016). The IL-6 concentration in PRGF was similar to the concentrations seen previously, although IL-1β was not found in PRGF, it was found in other PRPs with values similar to those identified here (Masuki et al., 2016). The presence of these and others inflammatory cytokines in the PRGF was confirmed and corroborated here. Moreover, the activation of microglia by cytokines was demonstrated due to the presence of cytokine receptors on microglia, which express mRNA transcripts for IL-1 receptor I and II, the IL-6, IL-8, IL-10, IL-12, IL-13, IL-15 receptors, and TNF receptors I and II (Lee et al., 2002). In addition, IL-6, IL-12 and TNFα can activate microglia (Luo and Chen, 2012) and thus, the cytokines present in PRGF can themselves trigger an inflammatory response. This activation would be independent of the cytokine secretion by the tissue exposed to the PRGF, as PRGF has been shown to induce the expression of cytokines like IL-1β, IL-6 or IL-10 (Mozzati et al., 2010). In addition, it has been suggested that the anti-inflammatory effect of PRGF is due to a high IL-4 content (Anitua et al., 2012), although the presence of this and other anti-inflammatory cytokines like IL-10 and IL-13 (Cavaillon, 2001) is weak relative to other cytokines that fulfill an opposite effect, such as the pro-inflammatory cytokine IL-1β.

In order to dampen microglial migration in the retinal explants, the PRGF was heat-inactivated prior to exposure to the explants (Anitua et al., 2014a). We also used Dex to inhibit inflammation (Tsurufuji et al., 1984) and we used antibodies against pro-inflammatory cytokines to block their action. The cytokines selected to mimic a pro-inflammatory environment in the organotypic cultures were the three major pro-inflammatory cytokines: IL-1β, IL-6, and TNFα (Taki et al., 2007; Zhang and An, 2007). It has been demonstrated that reactive microglia increase their expression and the release of these cytokines in retinal organotypic cultures (Madeira et al., 2015b). Moreover, the presence of antibodies against these three cytokines is sufficient to significantly diminish the number of activated microglia. However, the neutralization of the pro-inflammatory cytokines by antibodies did not completely suppress microglial migration, which could be due to the fact that these antibodies are from other species (e.g., goat, mouse and rabbit) and that this might provoke an immune response. Alternatively, Dex and heat-inactivated PRGF can mildly, though not entirely, reduce microglial migration. In addition, the presence of pro-inflammatory cytokines in the cultures does not mimic the effect of PRGF. These results suggest that there are other factors in addition to the cytokines implicated in the activation of microglial migration. It is known that heat-inactivation maintains the biological activity of PRGF, yet it fully reduced complement activity and significantly dampened the presence of IgE (Anitua et al., 2014a). However, in addition to cytokines, IgM and IgG are also present in the heat-inactivated PRGF (Anitua et al., 2014a), and IgG increases microglial activation due to the expression of high affinity IgG receptor (FcαRI: Orr et al., 2005). Thus, complement and immunoglobulins, as well as cytokines, could participate in the activation of the retinal inflammatory response. Moreover, Dex did not totally block the effect of the cytokines in the medium (Pereiro et al., 2018), confirming that microglial activation is triggered by different factors.

By contrast, microglial migration is higher when the PRGF is heterologous (the donor is from a different animal from that which donates the retina) in comparison to the migratory effect when the PRGF is autologous. In addition, human PRGF (when the donor is from a different species) induces stronger microglial activation than the pig PRGF in the porcine retina and this could be due to the presence of the pro-inflammatory cytokine IL-8, which is significantly more abundant in the human PRGF. Moreover, the presence of other receptors in microglial cells, such as toll like receptors (TLR) that respond to self and non-self activators (Shastri et al., 2013), could be implicated in the activation of microglia when the PRGF is not autologous or it is from another species. Therefore, patients suffering from autoimmune diseases, or patients in which their own PRGF is not as effective as expected, should receive PRGF from a donor. Moreover, this heterologous PRGF must be inactivated or combined with an anti-inflammatory treatment in order to mitigate the inflammatory response that might be initiated.

In terms of gliosis, it was suggested that PRGF decreases astrocyte reactivity (Anitua et al., 2013; Anitua et al., 2014b). However, we found that human PRGF initiated an inflammatory response activating porcine microglial migration, inflammation that could activate astrocytes and Müller cells. In gliosis, macroglial cells can divide and become hypertrophic, producing long, thick processes, as well as overexpressing GFAP (Lee et al., 2002; Vecino et al., 2016). Nevertheless, in our model of retinal explants astroglia are activated *per se* and an increase in gliosis was not evident in astrocytes in the presence of PRGF. However, a mild increase in Müller cell gliosis was seen in the presence of PRGF. In summary, some heterogeneity in GFAP expression was found in the organotypic cultures, regardless of PRGF. It is known that biomechanical tension is a vital factor in maintaining retinal tissue integrity in organotypic cultures, and stretched retinal explants displayed no signs of gliosis, as well as increased neuronal survival and preservation of retinal architecture (Taylor et al., 2013). Therefore, some variability in the tensile strength of explants may explain the results obtained.

The proliferative effects of PRGF were first demonstrated in dentistry, oral implantology, orthopedics, sports medicine and the treatment of skin disorders (Anitua et al., 2010). This proliferative effect is due to the growth factors in PRGF, such as IGF-1, PDGF or FGF, powerful stimulators of cell replication/proliferation (Anitua et al., 2009; Vahabi et al., 2015). In ophthalmology, intravitreal injection of PRP induces proliferation of retinal fibroblast-like cells and it was used as a model of proliferative vitreoretinopathy (Pinon et al., 1992). Moreover, different platelet preparations produce similar proliferative effects on immortalized human Müller cells (MIO-M1), in addition to stimulating their migration (Burmeister et al., 2009). However, our results do not demonstrate that PRGF induces the proliferation of glial cells. This could be due to proliferation being inhibited by cell-cell contact, which might occur in many cells (Ribatti, 2017), as the retinal explant used here preserves retinal architecture and the cells are in contact in a physiological manner. However, in cultured Müller cells, the application of PRGF increases the Müller cell number (Ruzafa et al., 2021). It is possible that we could not detect Müller proliferation in our explants due to the preservation of retinal structure, as we have shown *in vitro.* It may be possible that Müller glia proliferation underlies the structural restauration of the retina in patients with macular hole after injecting autologous PRGF. On the other hand, microglia reaction is beneficial by the acquisition of new functions before the alteration of their homeostatic roles (Sierra et al., 2013; Hemonnot et al., 2019). These beneficial properties of microglia could positively affect the restoration of the macular hole in patients.

In the CNS, microglia immediately migrate to sites of tissue damage (Nimmerjahn et al., 2005). By contrast, macroglia do not migrate but they may die in the focal point of severe lesions, or they may become reactive and hypertrophic, in some cases proliferating (Bardehle et al., 2013; Burda and Sofroniew, 2014). PRGF appears to promote microglial migration with only mild signs of gliosis and with no increase in the rate of glial cell proliferation, and interspecific PRGF induces mild signs of inflammation, which could be easily reverted by inactivating the PRGF or combining it with anti-inflammatory drugs or antibodies against cytokines. However, *in vivo* studies will be required to confirm these results as the behavior of the cells could differ.

Previous studies suggested that PRGF induces neuroprotection by activating anti-apoptotic PI3K/Akt signaling and/or diminishing caspase-3 levels (Anitua et al., 2013). Moreover, it has been suggested that PRGF induces neuroprotection in association with a down-regulation of the activated microglial cell number and a significant decrease of pro-inflammatory mediators (Anitua et al., 2015a). Studies of other neurodegenerative diseases, such as AD (Anitua et al., 2013; Anitua et al., 2014b) and Parkinson’s disease (Anitua et al., 2015a), show that cell survival was enhanced by PRGF in primary neuronal cultures, and the number of degenerating neurons was reduced. However, PRGF activated microglia here and we did not find changes in RGC survival. The concentrations of PRGF used here were 10%, as used when studying the effects of human PRGF in mouse models (Anitua et al., 2013; Anitua et al., 2014b). Thus, the concentration and interspecies interactions here are unlikely to affect neuroprotection. By contrast, it was suggested that thrombin-activated PRP releases glutamate, and when it is applied to neuronal cultures it is neurotoxic (Bell et al., 2014). Moreover, some of the morphogens or growth factors present in PRP, like PRGF, could have an adverse effect on neurons, such as TGF-β1 that exerts negative effects on axon growth in rat brain-spinal cord co-culture (Takeuchi et al., 2012). Such results could explain why we did not find a neuroprotective role of PRGF in the retinal explants.

Although PRGF has been successfully used in different medical and surgical specialties, and there is extensive data indicating that PRP induces tissue regeneration, many of these studies are not sufficiently rigorous or controlled, and their data is often limited. Indeed, other studies have produced contrasting results (Kuffler, 2015). Currently, autologous PRP injections have been used as pilot treatments for some retinal lesions (Nadal et al., 2012; Arias et al., 2019). Although structural recovery of the retina may be achieved (for instance closure of a macular hole), a functional recovery might be limited by the potential increase in inflammatory reaction which may have deleterious for the cells and be responsible for several complications like focal macular epithelial pigmentary hypertrophy, development of epiretinal membrane and cataract progression among other (Minihan et al., 1997; Cheung et al., 2005; Nugent and Lee, 2015). Therefore, the impact of PRGF is not fully understood and the applications of PRGF in ophthalmology must be analyzed in more detail. Differences in the levels of the factors present in PRGF and in other blood-derived products may explain the variability in the results obtained in different studies (Vahabi et al., 2015), as evident through the significant differences in the cytokine concentrations between samples observed here. Thus, further animal and clinical studies should be performed to clarify the properties of PRGF.

## Conclusion

PRGF has been used in ophthalmology to treat persistent macular holes and recurrent retinal detachment, although the mechanisms through which PRGF works in the CNS remain unclear. In retinal organotypic cultures, PRGF induces microglial activation, an inflammatory response that may be due to the presence of inflammatory cytokines. However, we cannot rule out the possible positive effect of microglial cells in repairing the retina, as suggested. Autologous PRGF induces weaker signs of inflammation that heterologous PRGF and that from other species, and this microglia activation may be mitigated. Moreover, PRGF does induce partial gliosis, although it does not appear to induce glial proliferation or RGC neuroprotection. Thus, while PRGF could be a good candidate to stimulate and accelerate tissue regeneration, more studies will be necessary to clarify its effects on the nervous and immune systems.

## Data Availability

The raw data supporting the conclusion of this article will be made available by the authors, without undue reservation.
